# Bone Metastasis from Renal Cell Carcinoma

**DOI:** 10.3390/ijms17060987

**Published:** 2016-06-22

**Authors:** Szu-Chia Chen, Po-Lin Kuo

**Affiliations:** 1 Graduate Institute of Clinical Medicine, College of Medicine, Kaohsiung Medical University, Kaohsiung 807, Taiwan; scarchenone@yahoo.com.tw; 2 Division of Nephrology, Department of Internal Medicine, Kaohsiung Medical University Hospital, Kaohsiung Medical University, Kaohsiung 807, Taiwan; 3 Department of Internal Medicine, Kaohsiung Municipal Hsiao-Kang Hospital, Kaohsiung Medical University, Kaohsiung 812, Taiwan; 4 Faculty of Medicine, College of Medicine, Kaohsiung Medical University, Kaohsiung 807, Taiwan; 5 Institute of Medical Science and Technology, National Sun Yat-Sen University, Kaohsiung 804, Taiwan

**Keywords:** renal cell carcinoma, bone metastasis, molecular mechanisms, bone turnover markers, therapies

## Abstract

About one-third of patients with advanced renal cell carcinoma (RCC) have bone metastasis that are often osteolytic and cause substantial morbidity, such as pain, pathologic fracture, spinal cord compression and hypercalcemia. The presence of bone metastasis in RCC is also associated with poor prognosis. Bone-targeted treatment using bisphosphonate and denosumab can reduce skeletal complications in RCC, but does not cure the disease or improve survival. Elucidating the molecular mechanisms of tumor-induced changes in the bone microenvironment is needed to develop effective treatment. The “vicious cycle” hypothesis has been used to describe how tumor cells interact with the bone microenvironment to drive bone destruction and tumor growth. Tumor cells secrete factors like parathyroid hormone-related peptide, transforming growth factor-β and vascular endothelial growth factor, which stimulate osteoblasts and increase the production of the receptor activator of nuclear factor κB ligand (RANKL). In turn, the overexpression of RANKL leads to increased osteoclast formation, activation and survival, thereby enhancing bone resorption. This review presents a general survey on bone metastasis in RCC by natural history, interaction among the immune system, bone and tumor, molecular mechanisms, bone turnover markers, therapies and healthcare burden.

## 1. Introduction

Renal cell carcinoma (RCC) is a group of malignancies arising from the epithelium of renal tubules [1]. It is the most common type of kidney cancer in adults, accounting for 90%–95% of cases. It is subdivided into different histopathologic entities, with clear cell RCC as the most frequent. Initial treatment is usually either a partial or complete removal of the affected kidneys [2]. When metastasis occurs in RCC, the most common spread is to the lungs, bones, lymph nodes, liver, adrenal glands and brain. The median survival of patients with metastatic RCC (mRCC) is approximately eight months [3], with 50% of patients who present with metastases dying within the first year. Only 10% survive longer than five years [4].

Previously, immunotherapy with cytokines was the standard treatment. Subsequently, targeted agents, such as vascular endothelial growth factor-tyrosine kinase inhibitors (VEGF-TKIs) and inhibitors of mammalian target of rapamycin, showed significantly better responses and progression-free survival [5]. In terms of disease-specific survival for *de novo* mRCC between 1992–2004 (pre-targeted therapy) and 2005–2009 (era of targeted therapies), there was an improvement from 13 to 16 months [5].

The prognosis of patients with mRCC has improved with the introduction of molecular-targeted therapy. Motzer *et al.* [6] reported that the median overall survival was greater in the sunitinib group (26.4 months) than in the interferon-α (IFN-α) group (21.9 months), but the prognosis remained poor. Moreover, RCC is characterized by a high degree of resistance to chemotherapy. For instance, IFN-α and interleukin-2 as an immunotherapeutic treatment achieve complete or partial response in only 10%–20% of patients [7,8,9].

Common among patients with RCC, bone metastasis accounts for one-third of patients with metastatic disease [10,11]. This review aims to present a general survey on bone metastasis in RCC by natural history, interaction among the immune system, bone and tumor, molecular mechanisms, bone turnover markers, therapies and healthcare burden.

## 2. Natural History of Bone Metastasis in RCC

Bone metastases in RCC are mainly osteolytic, thereby decreasing bone integrity, inducing bone pain and resulting in significant morbidity for patients with associated skeletal-related events (SREs). Such morbidities include pathologic fractures, bone pain requiring radiotherapy, impending fracture needing surgical intervention, spinal cord and nerve root compressions and hypercalcemia [12]. The SREs can significantly decrease functional independence with loss of autonomy and impair quality of life [13].

In a study of 803 patients with mRCC treated in a tertiary center between 1998 and 2007, Woodward *et al.* [11] found that 32% of patients presented with or later developed bone metastases. The mean number of SREs experienced by those with bone metastasis over the disease course was 2.4. Furthermore, 80% received radiotherapy for bone pain, and 28% experienced spinal cord/nerve root compression [11].

Santini *et al.* [14] also investigated the natural history of bone metastasis in RCC and found that the median time to bone metastasis was 25 months for patients without bone metastasis upon diagnosis. At the Memorial Sloan Kettering Cancer Center, the median time to diagnosis of bone metastasis was 24 months for good risk, five months for intermediate risk and zero months for poor risk. In addition, 71% of patients experienced at least one SRE. The median time to the first, second and third SRE was 2, 5 and 12 months, respectively [14]. Table 1 shows the median survival time of RCC patients according to bone involvement time [11,14]. However, these studies are retrospective [11,14]. Prospective studies are still warranted to address specific issues.

Identifying RCC patients with bone metastasis for aggressive treatment is therefore important for attenuating disease and improving outcomes. Aside from tumor stage and grade, time from nephrectomy to bone metastasis (TTBM) is also considered important for prognostication. In the study by Santoni, RCC patients with TTBM >5 years had longer overall survival than those with TTBM <1 year or between one and five years (22 *vs.* 13 *vs.* 19 months) [15], suggesting that TTBM should be considered in order to optimize the management of RCC with bone metastasis.

Late recurrence, or recurrent disease noted ≥10 years after nephrectomy, is considered unusual for RCC [16]. The study by Miyao *et al.* revealed a late recurrence rate of 10.5% and 21.6% at 15 and 20 years, respectively [17]. Moreover, 6.4% of patients had late recurrence in 44 sites, including the lungs (36.4%), kidneys (25%) and bone (13.6%), followed by the brain, pancreas, adrenal glands, lymph nodes and liver. Their results underscore the need for RCC surveillance more than 10 years after nephrectomy. Symptom-based follow-up is likewise important, especially for bone metastases.

## 3. Interactions among the Immune System, Bone and Tumor

Bone metastases are a common cause of morbidity in patients with different types of cancer. Such metastases are classified as osteolytic, osteoblastic or mixed. Osteoclasts are mainly responsible for bone destruction in osteolytic lesions, even if their activation varies depending on the tumors. They are multi-nucleated cells of hematopoietic origin in bone, and their main function is the resorption of mineralized bone matrix [18]. They also create a microenvironment with the underlying bone matrix to form a specialized structure called a sealing zone.

Tumor cells interact with the bone microenvironment and induce immune cell activation that releases factors promoting bone metastases. Elucidating the interactions between the immune system and solid tumors in the formation of bone metastasis is imperative for developing effective treatment.

### 3.1. Interplay between Bone and the Immune System

An important molecular association between the immune system and bone is expressed by the receptor activator of nuclear factor NF-κB ligand (RANKL), a ligand for the receptor activator of nuclear factor NF-κB (RANK), and the natural decoy receptor osteoprotegerin (OPG). The membrane RANKL is represented by osteoblasts/stromal cells, and the soluble RANKL is excreted by active T cells; otherwise, the receptor RANK is displayed on osteoclast precursors, as well as on tumor cells [19,20,21]. The ratio of RANKL to OPG in serum has been considered to be the key factor in determining osteoclast activation at the bone level, with a higher serum RANKL/OPG ratio being an index for the upregulation of osteoclastogenesis [22]. The RANKL production by active T cells can directly regulate osteoclastogenesis and bone remodeling. Besides, RANKL explains some different pathological conditions, such as systemic and local bone loss in cancer. Many immune factors, including co-stimulatory receptors, cytokines, such as interferon-γ and tumor necrosis factor (TNF), and T and B lymphocytes play a key role in the regulation of bone cell development and bone turnover, as well as in the pathogenesis of bone disease [23].

Mikami *et al.* [24] investigated the role of the RANKL-RANK-OPG system in RCC and found that RANKL and RANK expressions positively correlate with the primary tumor stage. Both RANKL and RANK are also highly expressed in mRCC in the bone and other organs. Furthermore, elevated RANKL and RANK expressions are significant predictors of recurrence, bone metastasis and poor prognosis. Hence, by stimulating cancer cell migration and osteoclast activation, the RANKL-RANKL-OPG system may be involved in the metastasis of RCCs [24].

A study by Beuselinck [25] involving 129 RCC patients treated with anti-VEGFR-TKIs revealed that an elevated RANK/OPG ratio was associated with shorter time to bone metastasis, shorter median overall survival from initial diagnosis and shorter median progression-free survival. The results suggested that the RANK/OPG ratio in RCC might be associated with bone metastasis and prognosis in patients treated with anti-VEGFR-TKIs [25]. Nonetheless, further validation is warranted.

### 3.2. Interplay between Bone and RCC

The local interactions between tumor cells and bone form a vicious cycle underlying the development of skeletal metastases [26]. The bone marrow is favored by certain tumor cells that have a biological proclivity for it; for instance, bone marrow produces factors like CXCL12, which has a chemotactic role on cancer cells. On the other hand, cancer cells express the chemokine receptors, CXCR4 and CXCR7 [27]. Activated osteoclasts resorb bone and release growth factors, like bone morphogenetic proteins, transforming growth factor-β (TGF-β), insulin-like growth factor and fibroblast growth factor, and others that stimulate metastatic tumor cell growth [28]. In turn, cancer cells secrete prostaglandins, parathyroid hormone, parathyroid hormone-related peptide (PTHrP), activated vitamin D, interleukin-6 and TNF. These increase RANKL expression on osteoblasts and bone marrow stromal cells and stimulate osteoclast number, survival and activity, promoting osteolytic metastases [18].

### 3.3. Interplay between the Immune System and RCC

The vicious cycle concept has been enlarged to include T cells as additional regulators of bone tumor growth [29]. Blocking osteoclast activity efficiently decreases the tumor burden and the associated bone lesions in immuno-compromised animals bearing human osteolytic cancers. Although anti-resorptive therapies efficiently block osteoclasts, some treated patients develop skeletal metastases within two years of treatment, suggesting that other cells modulate bone tumor growth [29]. These cells are T lymphocytes. Tumor cell-derived interleukin-6, interleukin-1 and TGF-β can drive T cell differentiation towards a Th17 secretory helper cell phenotype that can induce RANKL production and osteoclasts activation via interleukin-17 production [30].

## 4. Molecular Mechanisms of RCC

### 4.1. The Role of TGF-β in RCC Bone Metastasis

Bone is a reservoir for numerous growth factors that make it a favorable environment for tumor growth [31]. Recent studies on breast cancer suggest that TGF-β1 drives a relentless cycle of tumor growth and bone destruction, where metastatic cells are stimulated by TGF-β1. This results in tumor cell secretion of factors that promote bone resorption, which, in turn, frees active TGF-β1 that fuels the cycle [31,32].

Kominsky *et al.* [33] investigated the role of TGF-β1 in RCC bone metastasis and found that TGF-β1-stimulated RCC bone metastasis cells played a role in promoting tumor growth and osteolysis *in vivo*, perhaps by initiating tumor-promoting paracrine interactions between tumor cells and the bone microenvironment. The results suggest that inhibiting TGF-β1 signaling may be useful against RCC bone metastasis [33].

### 4.2. The Role of TGF-α/Epidermal Growth Factor Receptor Signaling

The epidermal growth factor receptor (EGF-R) and its ligands, epidermal growth factor (EGF) and TGF-α, are overexpressed in human RCC compared to normal renal tissue [34]. Produced by RCC cells, TGF-α stimulates cell and endothelial cell proliferation. Together with EGF, it is a strong inducer of VEGF. The EGF-R signaling mechanisms are associated with the development and progression of RCC metastasis [35], but their importance in bone metastasis has not been demonstrated. Weber *et al.* [36] demonstrated that the expression of factors in the TGF-α/EGF-R cascade was elevated in human RCC bone metastasis tissues. The TGF-α, EGF, EGF-R and activated EGF-R were highly expressed in most tissues [36]. Takahashi *et al.* [37] also suggested that TGF-α and EGF might stimulate bone resorption by increasing the proliferation of osteoclast precursors, leading to more osteoclasts.

### 4.3. The Role of Insulin mRNA Binding Protein-3

Insulin mRNA binding protein-3 (IMP3) is an onco-fetal mRNA-binding protein recently described as an independent prognostic marker for distant metastasis in RCC and is associated with poorer survival [38]. In hepatocellular carcinoma, IMP3 has been associated with cell motility and trans-endothelial migration [39]. A member of the highly conserved protein family associated with mRNA transport, translation and turnover, IMPs can modulate cell proliferation, adhesion, migration and invasion [40]. Its expression is almost exclusively limited to embryonic development, and it is undetectable in most adult tissues. However, it has recently been found to be significantly expressed in malignant adult tissue, including RCC [41].

Xie *et al.* [42] assessed the correlation between computed tomography vascularity, and IMP3 and demonstrated an association between high IMP-3 expression and RCC bone metastasis in *in situ* and cell line studies. Nonetheless, more investigations on the diagnostic potential of biomarkers for RCC bone metastasis and the functional significance of IMP-3 in RCC vascularity and tumor progression are warranted.

### 4.4. The Role of Cadherin-11

For successful bone metastases, many alterations in tumor cells, including altered expression of adhesion factors, are needed. The adhesion molecule cadherin-11, a calcium-dependent cell-cell adhesion molecule and mesenchymal marker originally identified from mouse osteoblasts, is the most abundant cadherin present in human osteoblasts [43,44]. Recent studies demonstrate its essential roles in the formation of bone metastasis in prostate and breast cancer [45,46].

Satcher *et al.* [47] explored the possible mechanisms of homing/retaining RCC cells to bone and the subsequent proliferation for RCC cells to colonize bone. Compared to parental, liver or lymph node-derived cells, bone metastasis-derived 786-O cells (Bo-786-O) had significantly increased cadherin-11. While parental and Bo-786-O cells had similar proliferation rates, Bo-786-O cells also had increased migration. On the other hand, knockdown of cadherin-11 by shRNA reduced the migration rate of Bo-786-O cells, suggesting that cadherin-11 may contribute to the increased migration of bone-derived cells. Immuno-histochemical analysis of cadherin-11 expression in an array of human renal carcinoma tissues demonstrated that the number of human specimens with positive cadherin-11 activity was significantly higher in tumors that metastasized to bone than in the primary tumors themselves. Together, these results suggest that cadherin-11 may play a role in RCC bone metastasis [47].

### 4.5. The Role of PTHrP

Known as the tumor-derived agent responsible for humoral hypercalcemia of malignancy, PTHrP is a polyprotein derived from normal and malignant cells [48]. It regulates cell growth, differentiation and death [49]. Burton *et al.* revealed that PTHrP regulated the proliferation of the RCC line [50], while Massfelder *et al.* [51] demonstrated that blocking PTHrP with antibodies or antagonizing the common parathyroid hormone (PTH)/PTHrP receptor decreased the *in vitro* expansion of human clear RCC by promoting cell death. By inducing cell death in nude mice, anti-PTHrP antibodies induced the complete regression of 70% of the implanted tumors. Moreover, the von Hippel–Lindau (VHL) tumor suppressor protein, which was a gatekeeper for clear RCC, negatively regulated PTHrP expression at the post-transcriptional level. These findings indicate that PTHrP is essential for clear RCC and may be a novel target for the VHL tumor suppressor protein [51].

About 40%–80% of human conventional RCC have a functional inactivation of the VHL tumor suppressor gene [52]. Talon *et al.* [53] evaluated whether blocking the PTHrP/PTH1 receptor system might have therapeutic value against RCC, independent of VHL status and PTHrP expression levels. In vitro, tumor cell growth and viability decreased by as much as 80% due to apoptosis in all cell lines. Exogenously-added PTHrP had no effect on cell growth and viability, but reversed the inhibitory effects of the antibody. The growth inhibition was reproduced by a specific PTH1 receptor antagonist in all cell lines. In vivo, the treatment of nude mice bearing the Caki-1 RCC tumor with the PTHrP antibody inhibited tumor growth by 80%, also by inducing apoptosis. This study therefore provides a rationale for evaluating the blockade of PTHrP signaling as a therapy for human RCC in a clinical setting [53].

### 4.6. The Role of Calcium/Calcium-Sensing Receptor

The effect of extracellular calcium on cells implies an activation of the CaSR, a G-protein-coupled receptor [54]. Highly expressed in healthy kidneys, CaSR has several functions, including the regulation of extracellular calcium concentration and inorganic phosphate homeostasis, mono- and di-valent cation transport, the acidification and concentration of urine and renin release [55,56]. When activated by enhanced extracellular calcium concentration, it coordinates cellular responses through various intracellular signaling pathways, thereby modulating cell proliferation, differentiation, migration and apoptosis [57]. Its expression correlates with the formation of bone metastases in breast cancer [58].

Joeckel *et al.* [59] assessed the effect of extracellular calcium concentration on mechanisms in RCC bone metastasis using CaSR. The CaSR expression was highest in specimens and cells of patients with bone metastases. Calcium treatment led to increased migration and proliferation of RCC cells in patients with bone metastases, indicating that RCC bone metastasis might be promoted by enhanced CaSR expression. Nonetheless, the role of CaSR as a prognostic marker must be evaluated by further prospective studies [59].

### 4.7. The Role of AKT/Integrin-α5 Signaling

In previous evidence, the phosphoinositide 3-kinase (PI3K)/protein kinase B (AKT) signaling pathway, which is engaged in the development and progression of many malignancies, may be disrupted by varying integrin signaling [60]. Primary RCC cells can recognize increased levels of pro-migratory and pro-adhesive factors, like fibronectin and collagen I. These are highly concentrated in bone tissue and can promote RCC bone metastasis. Aside from adherence to ECM compounds, increased integrin α5 levels and downstream signaling via AKT can help tumor cells and facilitate their migration to bone [61], suggesting that integrin α5 may be a prognostic marker of RCC bone metastasis. In other tumors, an integrin α5 inhibitor being tested as cancer therapy in a phase II trial prevented tumor cell invasion and metastasis [62,63].

### 4.8. The Role of Matriptase and MET

As a high-affinity receptor tyrosine kinase of hepatocyte growth factor (HGF), MET is a well-known multifunctional growth factor. The HGF/MET signaling axis may also be involved in tumor progression [64], as increased MET and HGF expressions and enhanced activation of pro-HGF are all seen in clear cell RCC [65,66]. Moreover, poor prognosis has been correlated to the overexpression of HGF, the cellular activator of pro-HGF and MET, indicating the importance of HGF-dependent MET activation in the progression of clear cell RCC. The study by Weber *et al.* [36] revealed a high MET expression in an RBM1 cell line, which was developed from a bone lesion in a patients with mRCC [67].

Through a systemic release of HGF, breast cancer plays a role in the formation of a pre-metastatic niche in the bone [68]. In turn, HGF contributes to the plasticity of bone metastasis by mediating a cross-talk within the metastasis microenvironment via the Wnt-β-catenin and Src tyrosine kinase network [68]. Akt activity is downstream of HGF/MET and is involved in tumor angiogenesis through increased secretion of vascular endothelial growth factor and by mediating the expressions of nitric oxide and angiopoietins [69]. Osteolytic bone metastasis depends on the balance among autophagy, anoikis resistance and ossification. Thus, the HGF signaling pathway may have an important role in bone colonization [70].

Matriptase, a type-2 transmembrane serine protease, is the most efficient known cellular activator of pro-HGF. It has been proposed to initiate signaling and proteolytic cascades through its ability to activate pro-urokinase and protease-activated receptor 2 [71]. Protease-activated receptor 2 is reportedly important for normal osteoblast and osteoclast differentiation [72] and, as a proteolytic target for Matriptase, may be strongly associated with it in osteoclasts of bone specimens. Lastly, Matriptase is reported to efficiently activate macrophage-stimulating protein and platelet-derived growth factors C and D [73]. 

Using nephrectomy specimens from 17 RCC patients with metastasis and bone metastases specimens from seven RCC patients after metastasectomies, Mukai *et al.* [74] demonstrated a high MET expression at the primary sites in eight (47%) nephrectomy specimens and in six (86%) bone specimens. Matriptase was expressed in six (35%) nephrectomy specimens and in all seven (100%) bone specimens. Matriptase was also strongly expressed in the osteoclasts of five bone specimens. Post-operatively, the overall survival rate was significantly higher in the MET^−^ group than in the MET^+^ group. The high MET and matriptase expressions in RCC cells with bone metastasis and the matriptase expression in osteoclasts imply their role in bone metastasis [74].

### 4.9. The Role of MicroRNAs

Specific miRNAs have been pointed out as potential diagnostic materials to distinguish the subtypes of RCC [75]. Especially in clear cell RCC, the most common subtype of RCC, miRNAs are deregulated [76]. One study of Wotschofsky showed that a total of 23 miRNAs were downregulated in metastatic tissue samples of clear cell RCC compared to normal tissue [77].

Heinzelmann *et al.* [78] also determined the role of miRNAs in mRCC. Comparing 14 miRNAs differently expressed in metastatic primary clear cell RCC and distant metastases to non-metastatic primary tumors revealed a strong correlation of miRNAs to progression-free- and cancer-specific five-year survival [78]. The miRNAs are deregulated in metastatic primary clear cell RCC and may be promising prognostic markers predictive of metastasis. Since alterations in miRNA expression characterize distant metastases to different sites, miRNAs may be a helpful predictive tool for follow-up and for personalized therapy selection.

### 4.10. Other Immunogenic Biomarkers

Paule *et al.* [79] identified a novel combination of biomarkers associated with the risk of bone metastasis. Among the gene transcripts studied, the overexpressions of VEGFR-1, VEGFR-2, hypoxia-inducible factor-1α, urokinase plasminogen activator and plasminogen activator inhibitor-1 in tumor tissues were significantly associated with bone metastasis [79]. Their study was a useful tool in managing RCC patients with bone metastasis, as the predictive markers could help identify subclinical disease, improve staging and guide treatment decisions.

Wang *et al.* [80] explored the expression levels of enhancer of zeste homolog 2 (EZH2), matrix metalloproteinase-2 (MMP2) and tissue inhibitor of metalloproteinase-2 (TIMP2) as determinants of RCC-associated bone metastasis. The EZH2 and MMP2 proteins were more expressed in tissues of patients with RCC bone metastasis. The TIMP2 promoter was also highly methylated in ACHN-BO5 cells, an ACHN cell subline with high potential of bone metastasis, compared to in ACHN cells. The upregulation of EZH2, MMP2 and TIMP2 expressions correlated with RCC metastasis to bone tissues *ex vivo* and *in vitro* [80].

**Figure 1:**
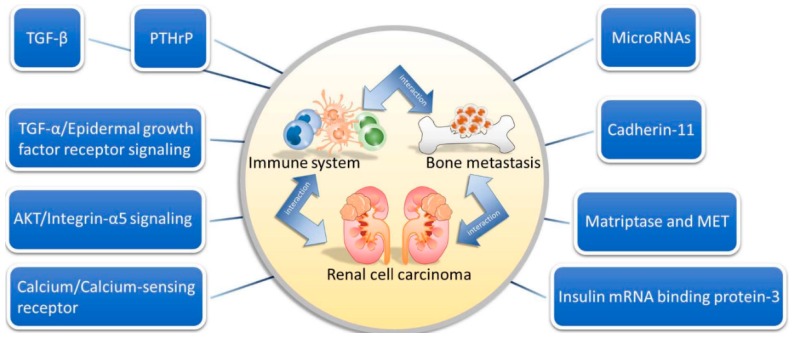
The molecular players in renal cell carcinoma.

Hence, there are many molecular mechanisms involved in the relationship between renal cell carcinoma, the immune system and bone metastasis, including TGF-β, TGF-α/EGF-R signaling, insulin mRNA binding protein-3 (IMP3), cadherin-11, PTHrP, calcium/CaSR, AKT/integrin-α5 signaling, matriptase, MET and miRNAs (Figure 1).

## 5. Diagnostic Value of Bone Turnover Markers in RCC Bone Metastasis

Bone metastases are characterized by increased osteoblastic and/or osteolytic processes, depending on the tumor type. In the metastatic process, more components of the osseous metabolism are released into the blood stream through increased metabolic activity and the overall destructive effect of metastasis [81]. These components include enzymes directly involved in the alteration processes, metabolites that are developed or bone matrix proteins that are released [82]. Patients with RCC bone metastases do not have significantly high levels compared to patients without metastases [83,84]. The concentrations of bone markers, like bone-specific alkaline phosphate, amino-terminally cross-linked telopeptides, tartrate-resistant acid phosphatase type 5b, osteopontin, OPG and RANKL, in patients with bone metastases also do not differ significantly from those of patients with RCC metastasis to other organs. Thus, the use of bone markers for diagnosing bone metastases in clinical practice has no clear scientific basis.

Klepzig studied if the bone turnover marker, procollagen type 1 amino-terminal propeptide (P1NP), could be an early predictor of bone metastases in patients with RCC and if chemotherapy could influence P1NP concentrations in patients with bone metastases [85]. The P1NP concentration was significantly higher among those with bone metastases than in those without. Patients treated with sorafenib had levels within the normal range. These suggest that P1NP may be a significant diagnostic marker for RCC bone metastases and may help in evaluating the progress of chemotherapy.

In addition, Alcaraz *et al.* [86] evaluated the correlation between bone turnover markers and outcomes in RCC patients who received zoledronic acid treatment. Patients with RCC who died or progressed had higher baseline β-CTX (β-carboxy-terminally cross-linked telopeptides) levels, while those with increased PINP levels developed new SRE. The β-CTX levels were associated with higher mortality and disease progression, while BAP was associated with increased risk of premature SRE appearance [86]. Both β-CTX and BAP can be considered complementary tools for predicting clinical outcomes in patients with RCC bone metastasis treated with zoledronic acid.

## 6. Therapy for RCC Bone Metastasis

Bone metastases are associated with pain, fracture, spinal cord compression, ineffective hematopoiesis and hypercalcemia of malignancy [12]. Treatment goals are the prevention of disease-related skeletal complications, pain palliation and the maintenance of quality of life.

### 6.1. Drug Therapy

#### 6.1.1. Zoledronic Acid

Zoledronic acid (2-(imidazol-1-yl)-hydroxy-ethylidene-1,1-bisphosphonic acid), a third-generation amino-bisphosphonate, is a potent inhibitor of osteoclast activity. It is widely used in the management of bone metastases from various malignancies, including RCC [87]. Its efficacy and safety have been established in three pivotal prospective, randomized controlled trials involving more than 3000 subjects [88,89,90]. Across a broad array of tumor types, it decreases the frequency of SREs, delays the time to a first SRE and reduces pain. It is more effective than pamidronate in breast cancer and is the only proven effective bisphosphonate for metastatic prostate cancer, lung cancer, RCC and other solid tumors.

Even as a single agent, zoledronic acid can decrease the risk of SREs and prolong the SRE-free survival in patients with RCC bone metastases. However, the objective response rate is quite low (7%), and more than half of the patients will eventually experience SREs [91]. Recent studies report that zoledronic acid potentiates the effects of radiotherapy on bone metastases from RCC [92,93]. Hence, a combination therapy may yield a significantly higher objective response rate and longer SRE-free survival compared to radiotherapy alone [92]. Zoledronic acid may directly radio-sensitize RCC cells at bone metastasis sites. Surveying the mechanism, Kijima *et al.* [94] found that zoledronic acid directly radio-sensitized RCC cells by potentiating the caspase-3-mediated apoptosis pathway. Post-transcriptionally, it downregulates the signal transducer and activator of transcription 1 (STAT1) expression, suggesting that STAT1 plays a key role. Further clinical and translational studies are warranted to explore these anti-tumor activities.

Broom *et al.* [95] evaluated the benefits of adding zoledronic acid to targeted therapy in patients with RCC bone metastasis. After 12 weeks, the median progression-free survival was 7.5 months on everolimus plus zoledronic acid and 5.4 months on everolimus alone. The median time to first SRE was 9.6 months on the combined therapy and 5.2 months on everolimus alone [86]. In this RCC population, the addition of zoledronic acid to everolimus significantly prolonged tumor control.

Nevertheless, toxicity issues must be considered. Acute-phase symptoms like flu-like symptoms are common, but can be easily managed with analgesics. Renal toxicity may also occur. In bone metastases arising from other tumors, this is normally managed by dose reduction, but may require special care in RCC [96]. Osteonecrosis of the jaw is a rare, but potentially serious event in patients with cancer who receive bisphosphonates, as it may be exacerbated by combined therapies [97].

#### 6.1.2. Denosumab

Research in bone biology has revealed a key pathway that regulates bone remodeling. Both OPG and RANKL are known essential mediators of osteoclast differentiation, activation and survival, leading to bone resorption [22]. The successful inhibition of RANKL to interrupt the “vicious cycle” of bone destruction and tumor proliferation points to a potential new treatment approach for bone metastases. In early studies, denosumab, a fully-human monoclonal antibody with high affinity and specificity for human RANKL, inhibits bone resorption in patients with advanced cancer [98,99,100], including those with failed bisphosphonate treatment [99].

In addition, Lipton *et al.* [101] evaluated the efficacy and safety of denosumab *versus* zoledronic acid from three pivotal, randomized, phase III trials involving more than 5700 patients with advanced cancer and bone metastasis [102,103,104]. Denosumab was better than zoledronic acid in delaying time to first on-study SRE by a median of 8.21 months and reduced the risk of a first SRE by 17%. Unlike zoledronic acid, denosumab did not require monitoring or dose modification based on renal status. It was also not associated with acute-phase reactions, although hypocalcemia was more common. Osteonecrosis of the jaw occurred at a similar rate. In patients with bone metastases from advanced cancer, denosumab was also superior to zoledronic acid in preventing SRE, with good safety and convenience. However, because of the design of these studies, the investigators were unable to collect data on patient preference for treatment, the impact of subcutaneous *versus* intravenous administration on patients’ quality of life and the time required for the different modes of administration. Denosumab was also not evaluated in patients with a baseline creatinine clearance of <30 mL/min. Zoledronic acid could not be used for patients with severe renal impairment.

#### 6.1.3. Cytotoxic T Lymphocyte Antigen-4 Antibody

Checkpoint receptors (CPRs) on cytotoxic T lymphocytes block co-stimulatory signals of immune activation after ligand binding, resulting in T cell anergy and immunosuppression. CTLA-4 is a CPR that ligates B7 molecules on antigen-presenting cells and inhibits T cell proliferation and function. Ipilimumab is a monoclonal antibody directed against CTLA-4 and is the first drug to improve overall survival of patients with previously-treated metastatic melanoma [105]. In a single institution phase II study of ipilimumab in mRCC unresponsive to interleukin-2, the higher dose group (3 mg/kg every three weeks) had five responses out of 40, while the lower dose group (3 mg/kg followed by 1 mg/kg every three weeks) had only one response out of 21. Blocking CTLA4 with ipilimumab induces cancer regression in some patients with mRCC, even if they are unresponsive to other immunotherapies [106].

#### 6.1.4. Programmed Death-1 Inhibitors

PD-1 is another T cell receptor expressed on activated, antigen-exhausted T cells that binds to its ligands to induce anergy. Nivolumab is a fully-humanized PD-1 blocking antibody that was tested in a large study that included 34 patients with mRCC [107]. Four of 17 patients (24%) treated with a dose of 1.0 mg/kg and five of 16 (31%) treated with 10.0 mg/kg had an objective response. Moreover, more than 50% of responders had responses lasting ≥1 year, including one responder with a complete response (6%). Nine patients (27%) had stable disease after 24 weeks.

Anti-PD-1 antibody produced objective responses in approximately one in 20%–25% of patients with non-small-cell lung cancer, melanoma or RCC [107]. In 2015, the results of a phase II study evaluating the efficacy of nivolumab in 168 patients with previously-treated mRCC [108] revealed no dose-response relationship for progression-free survival and a response rate of at least 20% in all doses. Despite unimpressive progression-free survival, the responses persisted for about two years, and there was better overall survival in doses of 2 and 10 mg/kg (25.5 and 24.7 months, respectively, *versus* 18.2 months for 0.3 mg/kg). In these three doses studied, nivolumab demonstrated anti-tumor activity with a manageable safety profile.

### 6.2. Non-Drug Therapy

Local treatment of metastases, including metastasectomy or radiotherapy, remains controversial in mRCC. Surgical resection is possible, but the accessibility and resectability of the metastases, as well as patients’ performance and co-morbidities are issues [109]. Since all patients who receive this treatment wind up in palliative care, the management goals must include timely pain control and mitigating complications, both of the treatment and of the disease process.

Even with advances in systemic treatment, radiation therapy (RT) is still a valid local, non-invasive treatment alternative to surgery and can be used for the palliation of painful bone metastases, spinal cord compression and brain metastases. Significant technological advances in RT, such as intensity-modulation RT, image-guidance RT, stereotactic body RT and stereotactic radiosurgery (SRS), have enabled the delivery of high doses, with accuracy within millimeters. Durable local control may exceed 90% with SRS, independent of histology, thereby providing patients with excellent palliation and long-term disease-free intervals [110,111].

Treatment for non-operative painful bone metastases has always been conservative: using radiotherapy, chemotherapy and analgesics [99]. More recently, minimally-invasive techniques, including ethanol, laser, microwave, cryo-ablation and radiofrequency ablation, have been used for painful bone lesions [112,113,114,115,116].

## 7. Healthcare Burden of SREs in RCC Bone Metastasis

With the increasing incidence of RCC and the advent of novel targeted therapies, the number of patients with RCC and bone metastasis is also increasing. Based on recent data, 85% of patients with RCC bone metastasis experience SREs at some point, with a mean of >2% of patients [11]. Furthermore, SREs are associated with a significant economic burden. In a study in the United States, the average cost of an SRE in patients with metastatic prostate cancer ranges from $11,800 for radiotherapy to $88,000 for surgery [117].

In the U.S. between 1998 and 2010, patients were treated for RCC in a weighted estimate of 144,899 hospital visits. Of the visits, 20.8% of patients had at least one SRE. The overall inflation adjusted mean costs associated with hospital visits of patients with RCC bone metastasis increased by 207% between 1998 and 2010, from $27,278–$56,554, while the mean costs associated with SREs increased from $33,979–$95,767. Conversely, the rates of SREs and SRE-associated mortality decreased significantly. This study demonstrates a decreasing prevalence and mortality in SRE-associated hospitalization of patients with mRCC. However, the related costs are increasing. These findings highlight the need for cost-effective management of mRCC, including preventing the loss of bone mass and the early detection of SREs in patients at risk. Earlier identification of bone disease through newer positron emission tomography and magnetic resonance imaging techniques may prompt for appropriate therapy [118].

## Figures and Tables

**Table 1:** Median survival time of RCC patients (Data from Ref. [11,14]).

Status	Median Survival Time
Never developed bone metastasis	13.3 months
Diagnosis with bone metastasis	10.6–12 months
Developed bone metastasis later	19.6 months
Developed bone metastasis only	22.6 months
After the first SRE	10 months

## Abbreviations

RCC	renal cell carcinoma
mRCC	metastatic RCC
VEGF-TKIs	vascular endothelial growth factor-tyrosine kinase inhibitors
IFN-α	interferon-α
SREs	skeletal-related events
TTBM	time from nephrectomy to bone metastasis
RANKL	nuclear factor NF-κB ligand
RANK	nuclear factor NF-κB
OPG	osteoprotegerin
TNF	tumor necrosis factor
TGF-β	transforming growth factor-β
PTHrP	parathyroid hormone-related peptide
EGF-R	epidermal growth factor receptor
EGF	epidermal growth factor
IMP3	insulin mRNA binding protein-3
Bo-786-O	bone metastasis-derived 786-O cells
PTH	parathyroid hormone
VHL	von Hippel–Lindau
CaSR	calcium-sensing receptor
PI3K	phosphoinositide 3-kinase
AKT	protein kinase B
HGF	hepatocyte growth factor
CTLA-4	cytotoxic T lymphocyte antigen-4
CPRs	checkpoint receptors
PD-1	programmed death-1 inhibitors
